# Unmet need for family planning services among young married women (15–24 years) living in urban slums of India

**DOI:** 10.1186/s12905-020-01010-9

**Published:** 2020-09-03

**Authors:** Kriti Yadav, Monika Agarwal, Mukesh Shukla, Jai Vir Singh, Vijay Kumar Singh

**Affiliations:** 1 Assistant Professor, Vir Chandra Singh Garhwali Government Institute of Medical Science and Research, Srinagar, Pauri Garhwal, Uttarakhand India; 2 Department of Community Medicine & Public Health, K.G. Medical University, Lucknow, UP India; 3 Principal, Hind Institute of Medical Sciences, Lucknow, UP India

**Keywords:** Determinants, Family planning, Slums, Unmet need, Uttar Pradesh, Young married women

## Abstract

**Background:**

NFHS-4 stated high unmet need for family planning (FP) among married women in Uttar Pradesh. Unmet need is highest among age groups: 15–19 and 20–24 years. Currently few data is available about unmet need for FP among vulnerable section of the community, i.e.15–24 year’s age group living in the urban slums. Therefore this study was conducted to assess the unmet need for FP services and its determinants among this under-privileged and under-served section of society residing in urban slums of Uttar Pradesh, India.

**Methods:**

Cross sectional study was conducted in the slums of Lucknow, India. One Urban-Primary Health Centre (U-PHC) was randomly selected from each of the eight Municipal Corporation zones in Lucknow and two notified slums were randomly selected from each U-PHC. All the households in the selected slums were visited for interviewing 33 young married women (YMW) in each slum, with a pre-structured and pre tested questionnaire, to achieve the sample size of 535. Analysis of the data was done using logistic regression.

**Results:**

The unmet need for family planning services among YMW was 55.3%. About 40.9% of the unmet need was for spacing methods and 14.4% for limiting methods. Important reasons cited for unmet need for family planning services were negligent attitude of the women towards family planning, opposition by husband or others, embarrassment / hesitation / shyness for contraceptive use, poor knowledge of the FP method or availability of family planning services. Among method related reasons health concerns and fear of side effects were frequently cited reasons. On multiple logistic regression: age, educational status, duration of marriage, number of pregnancies, knowledge of contraceptive methods, opposition to contraceptive use and contact with Auxiliary Nurse Midwife (ANM) showed independently significant association with unmet need for family planning services.

**Conclusions:**

Unmet need for family planning services is very high among the YMW of urban slums. The findings stress that program managers should take into cognizance these determinants of high level of unmet need for family planning among YMW and make intense efforts for addressing these issues in a holistic manner.

## Background

United Nations Population Division states that by 2050, approximately 66% of the globe’s population will live in urban areas [1]. The urban poor have higher fertility, high unmet need for family planning services and poor maternal health outcomes [1]. A range of factors that characterize urban poverty contribute to these poor reproductive health outcomes: unemployment, unsanitary and overcrowded living conditions, inadequate access to formal health services, gender-based violence and limited autonomous decision-making for women [1]. The urban poor therefore face vulnerabilities that can put them at the disadvantage compared to their rural counterparts [1]. Also, the unmet need for family planning has been reported to be the highest among women who are younger than 20 years of age, and lowest among women aged 35 and older; these differences being found to be widest in South Central Asia, including India [2]. Similar findings have also been reported in the studies done in South East Asia [3], South Africa [4] and other developing nations of the world [5].

**Fig. 1:**
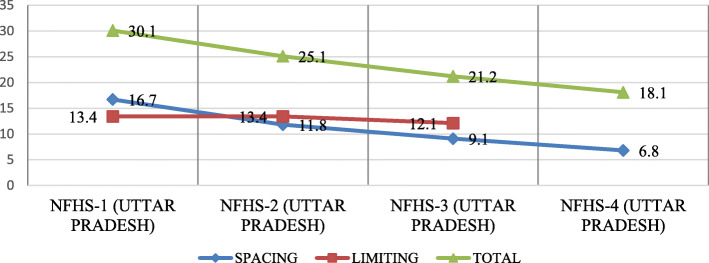
Trends of Unmet Need for Family Planning Uttar Pradesh (%) [6, 7, 12, 13]

The unmet need for family planning among all ages (15–45 years) married women in India is 12.9% [6] with unmet need for spacing and limiting being 5.6 and 7.2% respectively [6]. No significant decline in the unmet need for family planning has been observed over the past decades in the country [6, 7]. A high level of unmet need for family planning is seen among the age groups of 15–19 years and 20–24 years (27.1 and 22.1% respectively) [7]. Among all the states, Uttar Pradesh, with one sixth of India’s population (200 million) [8] shows an even worse picture with very high levels of unmet need of about 18.1% [6]. The state has an annual growth rate of about 16.5 [8], with total fertility rate (TFR) of 2.7 [6]. Also in the two target age groups i.e. 15–19 and 20–24 years the Age Specific Marital Fertility Rate (ASMFR) is reported to be the highest (271.0 and 383.9 respectively) [8]. In addition to that low Contraceptive prevalence rate (CPR) was also reported in these age groups (14.5 and 26.7% respectively) [7]. High fertility (2.96) and low contraceptive prevalence rate (58.2%) have also been reported in slums in comparison to non-slum areas (2.78 and 65.1%) [7]. State level data for slums in Uttar Pradesh shows a wide difference in unmet need between slum and non-slum areas (12.9 and 8.9% respectively) [7]. Other studies conducted in urban slums of Uttar Pradesh have also indicated a high unmet need among married women of 15–45 years age group [9–11]. About 44.5 million people reside in urban slums in UP (Census 2011) [8]. Mostly, young people migrating from rural areas in search of earning opportunities are settling in slums. Here, they not only lack basic amenities for living, they also do not have enough access to health services, which negates them from utilizing the facilities of health programs (Fig. 1).

Large population with relatively high fertility due to low use of contraceptives by this age group (15–24 years) and living in suboptimal conditions makes them the most preponderate group for family planning services from public health perspective [14]. To catch this young population it is imperative for policy makers and program managers to understand their need for FP services and factors influencing their needs for family planning. No such data is currently available in the country for this age group especially for the young married women living in the urban slums. As the reproductive health needs of the millions of urban poor cannot be ignored, therefore this study was conducted with an aim to assess the unmet need for family planning services among the currently married young women living in urban slums of Lucknow (Uttar Pradesh, India), the reasons for this unmet need for family planning services and the factors influencing it. This will help in delineating the individual, community and health services level factors that can be harnessed or changed to improve the contraceptive use and enable young women living in urban slums to satisfy their need for contraceptives at this stage of family building process.

## Objectives

1. To assess the unmet need for family planning services among the young married women living in urban slums of Lucknow, India.
2. To explore the facttors influencing the unmet need of family planning services among the young married women.

## Methods

### Study design

Cross sectional study.

#### Study settings

The study was conducted in the catchment slums of Urban-Primary Health Centres (UPHCs) of Lucknow. Lucknow, the capital of Uttar Pradesh is situated 123 m above sea level. It is situated between 26.30 and 27.10 North Latitude and 80.10 and 80.30 East Longitude. Lucknow has a population of 2,817,105, of which around 12.95% resides in slums with substandard living conditions [8]. Average literacy rate is 82.50% of which male and female literacy is 86.04 and 78.70%. Mean age of marriage is 22.9 years and median age of first live birth is 23.8 years [15]. Health services to the urban poor are provided through Urban- Primary Health Centres (U-PHCs), Bal Mahila Chikitsalays (BMCs), District Hospitals and plethora of private practitioners.

#### Study period

The study was conducted from August 2015 to July 2016.

#### Study universe

All the Young married women (15–24) living in urban slums.

#### Young married women [7, 16]

Currently married young women in the age group of 15–24 years. (Census- India, UN Secretariat).

#### Study population

Young married women (15–24) living in the urban slums of Lucknow.

#### Study unit

Young married woman (15–24) currently living in the urban slums of Lucknow for at least 6 months. However, women who were currently pregnant or had undergone hysterectomy/ bilateral oophorectomy or were divorced / separated / disserted from their husband were excluded from the study.

### Sample size determination

Sample size was calculated by the following formula, n = z^2^*p*(1-p)/ d^2^. Taking the unmet need of family planning services in Uttar Pradesh (p) as **14.6%** (AHS 2012–13) [15], an allowable error (d) of **3%** and the value of the standard normal variable at 0.05 (two sided) level of significance (z) as **1.96, the sample size was calculated to be 533.**

**Fig. 2:**
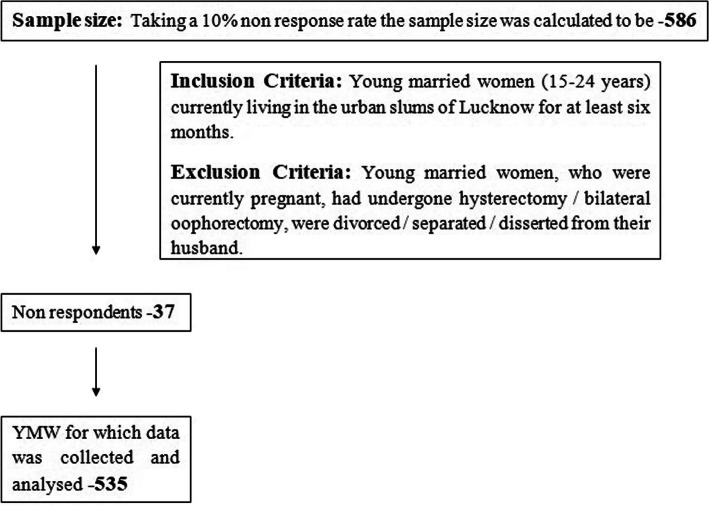
Strobe Diagram Depicting the participants through the study

Considering a 10% non-response rate; the final sample size was calculated as **586.** Excluding 37 non responding women; a sample of **535** was analyzed (Fig. 2).

### Sampling

**Fig. 3:**
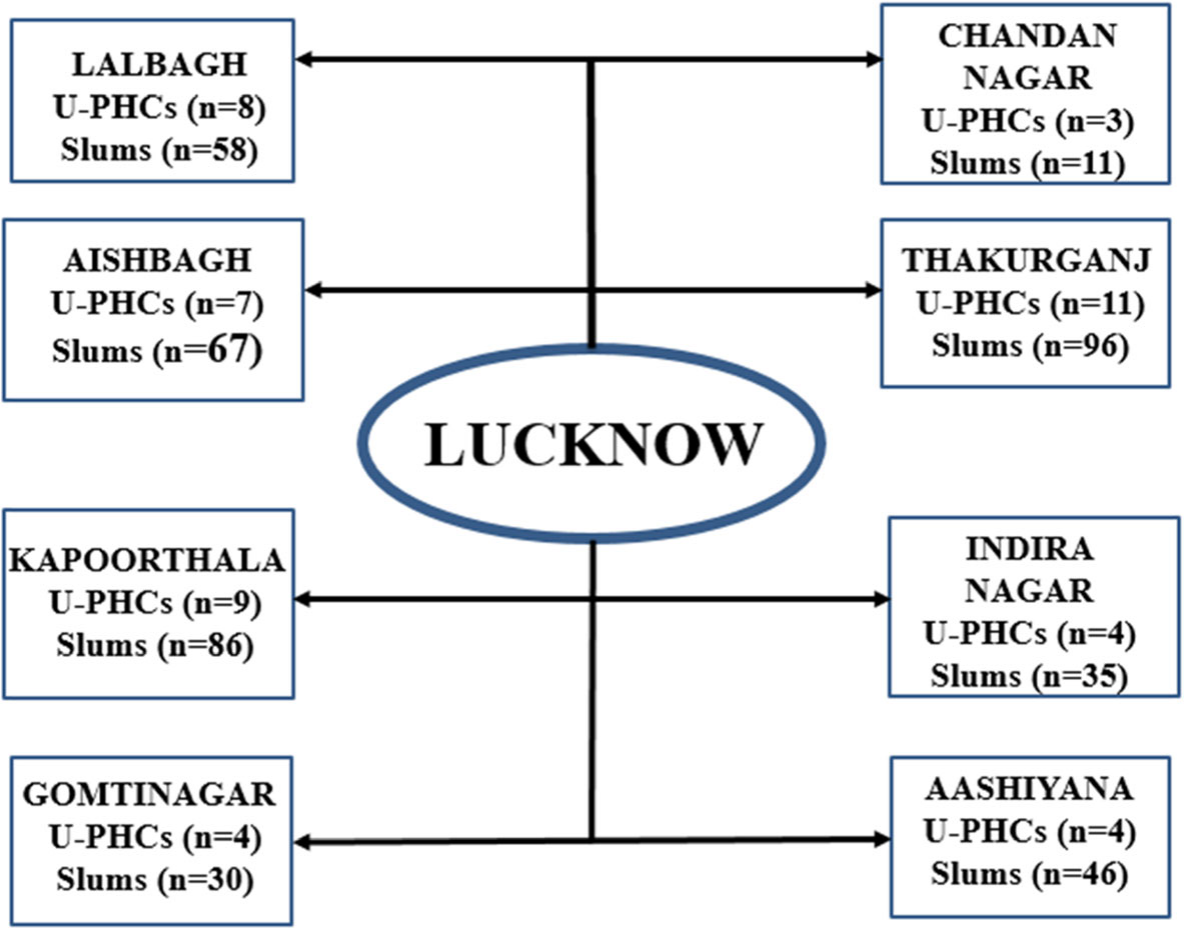
Total U-PHCs and Slums in the Municipal Corporation (Nagar Nigam) Zones OF Lucknow [17]

**Fig. 4:**
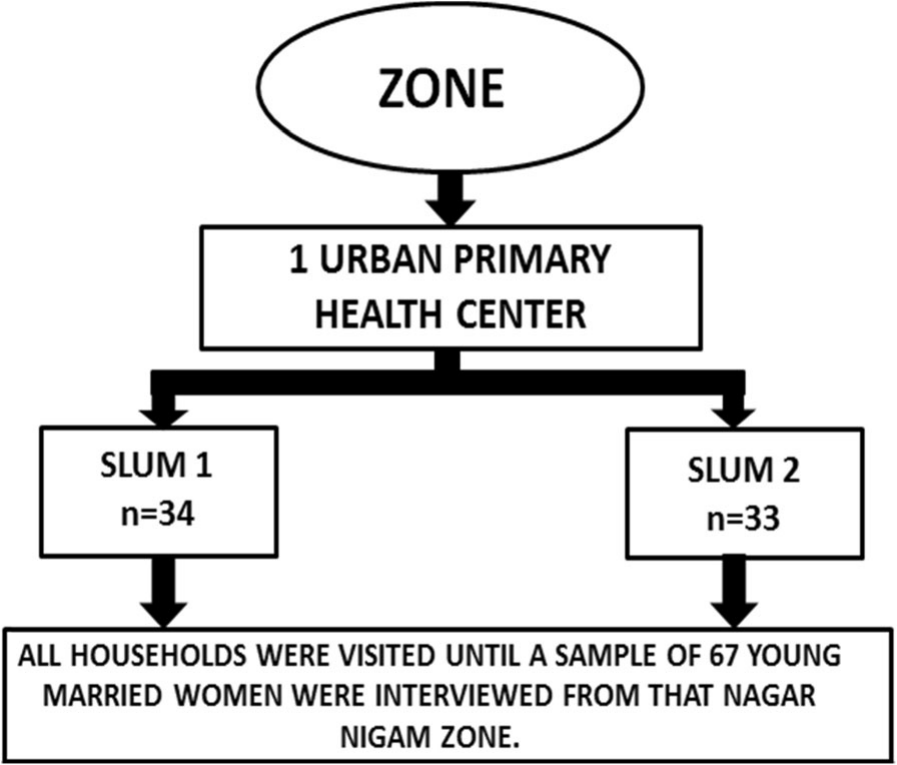
Illustration of Sampling Technique

To identify the eligible young women to be selected in the sample, a three staged random sampling technique was used. All the eight Municipal Corporation zones in Lucknow city were taken into consideration for selection of the study participants. One U-PHC was randomly selected from each Municipal Corporation zone. The zone wise list of slums notified by the Municipal Corporation was obtained from the Municipal Corporation office and two slums were randomly selected from each U-PHC. To obtain the desired sample from each slum, the total sample size was divided equally among the eight Municipal Corporation zones. A sample of 67 young married women was obtained for each zone. Thus, at least 33 young married women were selected from each slum (Figs. 3 and 4).

**Fig. 5:**
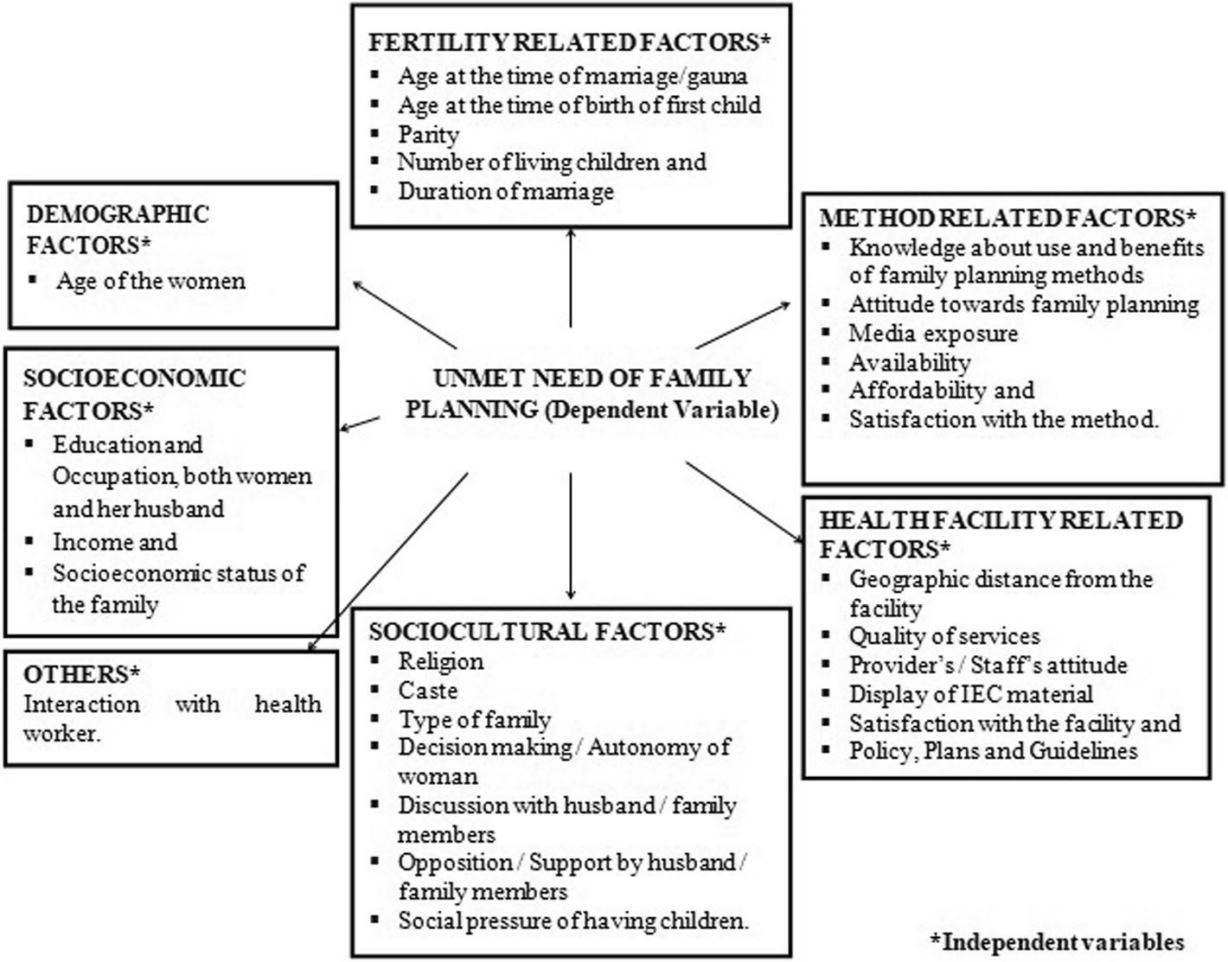
Conceptual Framework for the Determinants of Unmet Need of Family planning Services by young Married Women (Modified from study: Wulifan., et al., (2016) [27])

In each slum, the centre of the slum was arbitrarily identified and a sample of at least eight YMW was obtained from each direction. First household was randomly selected and all the households were visited until the desired sample was obtained for that slum.

## Operational definitions

### Slum [8]

The slum areas broadly constitute of:

- All specified areas in a town or city notified as ‘Slum’ by State/Local Government and UT Administration under any Act including a ‘Slum Act’.
- All areas recognized as ‘Slum’ by State/Local Government and UT Administration, Housing and Slum Boards, which may have not been formally notified as slum under any Act.
- A compact area of at least 300 populations or about 60–70 households of poorly built congested tenements, in unhygienic environment usually with inadequate infrastructure and lacking in proper sanitary and drinking water facilities.

### Catchment slum [18]

Slum in the geographic area defined and served by a health facility, which is delineated on the basis of such factors as population distribution, natural geographic boundaries, and transportation accessibility. By definition, all residents of the area needing the services provided by the health facility are usually eligible for them.

### Urban primary health Centre (U-PHC) [17]

Established by Government of India under the National Health Mission (NHM) to improve the health status of the urban poor particularly the slum dwellers and other disadvantaged groups by provisioning access to quality primary health care services along with strengthening the existing capacity of health delivery systems leading to improved health status and quality of life. A U-PHC caters to a population of 50,000. Currently, there are 52 U-PHCs in Lucknow.

### Family planning services [19]

It includes services that enable individuals to determine freely the number and spacing of their children and to select the means by which this may be achieved.

### Modern spacing methods [20]

Include contraceptive pills, condoms, injectables, intrauterine devices (IUDs / PPIUDs) and emergency contraception.

### Modern limiting methods [20]

Include male and female sterilization.

### Unmet need for modern family planningmethods [7]

The percentage of women of reproductive age who are not using any modern method of family planning but who would like to postpone the next pregnancy (unmet need for spacing) or do not want any more children (unmet need for limiting). The sum of the unmet need for limiting and the unmet need for spacing is the total unmet need for family planning.

### Unmet need for spacing [7]

It includes fecund women who are neither pregnant nor amenorrhoeic, who are not using any modern spacing method of family planning, and say they want to wait two or more years for their next birth. Also included in unmet need for spacing are fecund women who are not using any modern method of family planning and say they are unsure whether they want another child or who want another child but are unsure when to have the birth.

### Unmet need for limiting [7]

It refers to fecund women who are neither pregnant nor amenorrhoeic, who are not using any modern limiting method of family planning, and who want no more children.

### Met need for modern contraceptive methods [21]

Refers to those currently married women who want to space births or limit the number of children and are using modern contraceptive methods to avoid unwanted or mistimed pregnancies.

### Total demand for family Planning [21]

The total demand for family planning is the sum of unmet need and met need.

#### Tools of data collection

A pre-designed and pre-tested interview schedule [see Additional file 1] was used for data collection. Information was collected regarding: Bio-social characteristics, autonomy status of the women, knowledge regarding family planning, attitude towards contraceptive use, current use of contraceptives, factors favoring / limiting access and utilization of family planning services in young married women (Fig. 5). Religion was based on the belief system followed by the participant and caste / category on the official classification of the population of India [8]. Other Backward Class (OBC) is a collective term used by the Government of India to classify castes which are educationally or socially disadvantaged [8]. Scheduled Caste (SCs) and Scheduled Tribes (STs) are officially designated groups of people by the Constitution of India [8]. YMW above the age of 7 who can read and write in any language with an ability to understand was considered as literate [8]. Modified Kuppuswamy’s socioeconomic classification, a composite scale based on education, occupation of the head of the family and the monthly income of the family, was used to determine the socioeconomic status [22]. Autonomy [23] of the women was assessed in three dimensions of household decision making concerning money spent, health care and physical mobility and scored accordingly. Attitude of the women and her husband towards family planning was assessed from the responses given by the women on the pertaining questions. The schedule was pretested on a sample of 30 young married women living in urban slums of Lucknow. Inconsistencies and confusions in the pre-test exercise including the interview protocol were corrected before actual data collection. Result of pre-test was not included in final study. Completed schedules were checked weekly for consistency and completeness by the supervisors. The collected information was rechecked for its completeness and consistency before entering the data into a computer.

## Data management

### Data collection procedure

During the visits to the slums, the investigator approached the young married women fulfilling the inclusion criteria and after explaining then about the study; an informed consent was sought from them for their participation in the study. Complete confidentiality and anonymity of the respondents was maintained. Written and informed consent was taken. The study included 535 YMW who met the inclusion/exclusion criteria for the study.

### Data processing and analysis

Descriptive summary using frequencies, percentages, graphs and cross tabs were used to present study results. Univariate analysis was performed using binary logistic regression and the factors which were found significant during univariate analysis were forwarded to multiple logistic regression model in a step wise manner for calculation of Adjusted Odds Ratio. A *p* value < 0.05 was considered statistically significant.

## Results

**Fig. 6:**
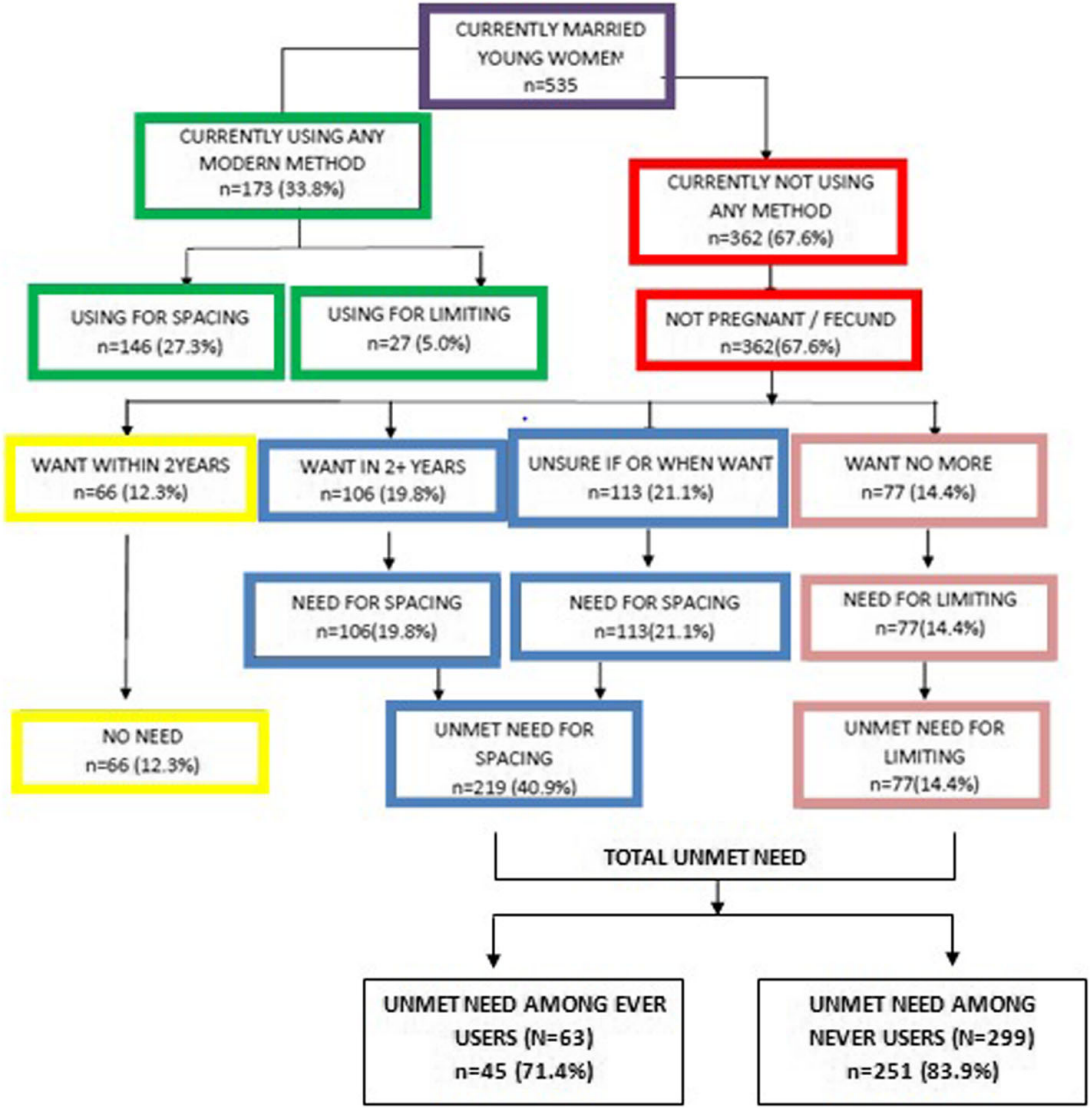
Need for Family planning Among Young Married Women

The total demand for family planning among the young married women living in urban slums of Lucknow was 87.6% (68.2% for spacing and 19.4% for limiting). Findings (Fig. 6) demonstrated considerably high unmet need for contraceptives among young married women in urban slums. It was found to be present in more than half (55.3%) of the young married women; of which in about 40.9% was for spacing methods and in 14.4% for limiting methods.

### Bio-social characteristics of women

The mean age of the study participants was found to be 21.28 ± 1.9 years. Most of the women were Hindu by religion (87.1%) and about 48.2% of them belonged to other backward classes (OBCs) (Table 1). About 18.7% of the study participants were illiterate. More (69.7%) women in the older age group (20–24 years) had high school and above level of education as compared to the younger age group (37.6%). Majority (81.5%) of the women in older age group were working outside home for money (Table 1). The mean duration of stay in the city was 2.72 ± 1.95 years.

**Table 1:** Percentage distribution of the Young Married Women by their age and bio-social characteristics

BACKGROUND CHARACTERISTICS	TOTAL WOMEN (***n*** = 535) (%)	AGE GROUP (Years)		***p*** VALUE
		15–19 (***n*** = 109) (%)	20–24 (***n*** = 426) (%)	
**Religion** [8]				
Hindu	466 [87.1]	90 (19.3) [82.6]	376 (80.7) [88.3]	0.113
Muslim	69 [12.9]	19 (27.5) [17.4]	50 (72.5) [11.7]	
**Caste / Tribe** [8]				
SC / ST	184 [34.4]	40 (21.7) [36.7]	144 (78.3) [33.8]	0.007**
OBC	258 [48.2]	61 (23.6) [56.0]	197 (76.4) [46.2]	
Others	93 [17.4]	8 (8.6) [7.3]	85 (91.4) [20.0]	
**Level of Education of the Respondent** [8]				
Illiterate	100 [18.7]	20 (20.0) [18.3]	80 (80.0) [18.8]	0.000***
Primary	141 [26.3]	48 (34.0) [44.0]	93 (66.0) [21.8]	
High School	220 [41.0]	34 (15.5) [31.2]	186 (84.5) [43.7]	
Intermediate and above	74 [13.8]	7 (9.5) [6.4]	67 (90.5) [15.7]	
**Level of Education of the Respondent’s Husband** [8]				
Illiterate	124 [23.1]	33 (26.6) [30.3]	91 (73.4) [21.4]	0.000***
Primary	140 [26.2]	41 (29.3) [37.6]	99 (70.7) [23.2]	
High School	208 [38.9]	31 (14.9) [28.4]	177 (85.1) [41.5]	
Intermediate and above	63 [11.8]	4 (6.3) [3.7]	59 (93.7) [13.8]	
**Employment Status of the Respondent**				
Unemployed	416 [77.8]	87 (20.9) [79.8]	329 (79.1) [77.2]	0.608
Employed	119 [22.2]	22 (18.5) [20.2]	97 (81.5) [22.8]	
**Socioeconomic Status** ^**#**^				
Lower and Upper lower	383 [71.6]	93 (24.3) [85.3]	290 (75.7) [68.1]	0.000***
Middle and Upper middle	152 [28.4]	16 (10.5) [14.7]	136 (89.5) [31.9]	
**Type of Family** [8]				
Nuclear	343 [64.1]	67 (19.5) [61.5]	276 (80.5) [64.8]	0.576
Joint	192 [35.9]	42 (21.9) [38.5]	150 (78.1) [35.2]	
**Duration of stay in the city (years)**				
< 1	186 [34.8]	90 (48.4) [82.6]	96 (51.6) [22.5]	0.000***
≥ 1	349 [65.2]	19 (5.4) [17.4]	330 (94.6) [77.5]	
**Duration of marriage (years)**				
**<** 1	86 [16.1]	56 (65.1) [51.4]	30 (34.9) [7.0]	0.000***
≥ 1	449 [83.9]	53 (11.8) [48.6]	396 (88.2) [93.0]	
**Parity**				
0	132 [24.7]	65 (49.2) [59.6]	67 (50.8) [15.7]	0.000***
1	157 [29.3]	28 (17.8) [25.7]	129 (82.2) [30.3]	
≥ 2	246 [46]	16 (6.5) [14.7]	230 (93.5) [54.0]	
**Knowledge of contraceptive methods**				
Yes	406 [75.9]	37 (9.1) [33.9]	369 (90.9) [86.8]	0.000***
No	129 [24.1]	72 (55.8) [66.1)	57 (44.2) [13.4]	
**Autonomy status** [23]				
Has no autonomy	145 [27.1]	71 (49.0) [65.1]	74 (51.0) [7.4]	0.000***
Has some autonomy	256 [47.9]	36 (14.1) [33.0]	220 (85.9) [51.6]	
Has autonomy	134 [25.0]	2 (1.5) [1.8]	132 (98.5) [31.0]	
**Media Exposure of message on Family planning on TV or Radio in the last 6 months**				
Yes	274 [51.2]	24 (8.8) [22.0]	250 (91.2) [58.7]	0.000***
No	261 [48.8]	85 (32.6) [78.0]	176 (67.4) [41.3]	
**Contact with health worker**				
Yes	114 [21.3]	9 (7.9) [8.3]	105 (92.1) [24.6]	0.000***
No	421 [78.7]	100 (23.8) [91.7]	321 (76.2) [75.4]	

#Modified Kuppuswamy Scale − 2016 [22] (Row %)[Column%] *p < 0.05, **p < 0.005, ***p < 0.001

The mean age at marriage and at the birth of first child was found to be 17.87 ± 1.85 and 19.23 ± 1.67 years respectively. More than half (59.6%) of the women in the age group of 15–19 years were nulliparous as compared to older age group (15.7%). About half (54%) of the older women had ≥2 children (Table 1). Teenage childbearing was reported to be about 8%.

Knowledge of contraceptives was significantly low (9.1%) in the younger women as compared to women in the older age group (90.9%) (Table 1). Autonomy in family and media exposure was significantly more in the women of older age group (Table 1). Contact with health worker was very low (8.3%) in the younger age group as well as with the women of older age group (24.6%) (Table 1). None of the young married women had received any education on family planning before marriage.

### Reasons for unmet need

More than two-third (69.2%) of the women in the study cited embarrassment / hesitancy / shyness to be a reason for unmet need for contraception. Knowledge of family planning methods and place where FP services are available was significantly low in 15–19 years age group in comparison to older age group (Table 2). About half (48.5%) of the older women had a negligent attitude towards adopting any family planning method and 45.6% of them faced opposition to contraceptive use; as a consequence of expectation for early child bearing; by the husband and family members. Health concerns and fear of side effects were frequently cited reasons of non use of contraceptives in the older age group (Table 2).

**Table 2:** Reasons for Unmet need for Family Planning services among Young Married Women in different age groups

REASONS	TOTAL WOMEN (***n*** = 296)	AGE-GROUP (Years)		***p*** VALUE
		15–19 (***n*** = 90)	20–24 (***n*** = 206)	
**Lack of Knowledge about Family Planning methods**	**163 (55.1)**	**72 (80.0)**	**91 (44.2)**	
Knows no method	134 (45.3)	71 (78.9)	63 (30.6)	0.000***
Don’t know how to use a method	29 (9.8)	1 (1.1)	28 (13.6)	0.003**
**Health Facility Related Reasons**	**165 (55.7)**	**69 (76.7)**	**96 (46.6)**	
No knowledge of place where Family Planning services are available	156 (52.7)	69 (76.7)	87 (42.2)	0.000***
Poor quality of services	7 (2.4)	0 (0)	7 (3.4)	0.722
Lack of access / Too far	2 (0.6)	0 (0)	2 (0.9)	0.322
Desired method not available	1 (0.3)	0 (0)	1 (0.5)	0.898
**Opposition to Use Family planning methods**	**111 (37.5)**	**17 (18.9)**	**94 (45.6)**	
Others oppose	58 (19.6)	13 (14.4)	45 (21.8)	0.358
Partner opposes	40 (13.5)	5 (5.5)	35 (17.0)	0.025*
Respondent herself opposes	37 (12.5)	3 (3.3)	34 (16.5)	0.006*
**Method Related Reasons**	**89 (30.1)**	**8 (8.9)**	**81 (39.3)**	
Health concerns	63 (21.3)	4 (4.4)	59 (28.6)	0.000***
Fear of side-effects	60 (20.3)	7 (7.8)	53 (25.7)	0.000***
Bad experience with previously used method	39 (13.2)	1 (1.1)	38 (18.4)	0.747
Inconvenience in using previously adopted method	20 (6.7)	0 (0)	20 (9.7)	0.492
Costs too much	3 (1.0)	0 (0)	3 (1.4)	0.822
**Fatalist approach**				
Upto God	39 (13.2)	4 (4.4)	35 (17.0)	0.001**
**Fertility Related**	**26 (8.8)**	**5 (5.5)**	**21 (10.2)**	
Currently Breastfeeding / Postpartum Amenorrhea	20 (6.8)	4 (4.4)	16 (7.8)	0.237
Infrequent sex / No sex	6 (2.0)	1 (1.1)	5 (2.4)	0.140
**Others**				
Embarrassment / Hesitation / Shy to talk about contraception	205 (69.2)	75 (83.3)	130 (63.1)	0.038*
Negligent attitude of the women towards family planning	119 (40.2)	19 (21.1)	100 (48.5)	0.000***
No opinion	2 (0.6)	0 (0)	2 (0.9)	–

#Multiple Responses (Column %) *p < 0.05, **p < 0.005, ***p < 0.001

### Factors influencing need of contraceptives among young married women:- Bivariate analysis

#### Bio-social factors

##### Age of the respondent:

Majority (90.9%) of the women in the age group of 15–19 had an unmet need for family planning services. The increase in age group was found to be significantly associated with decrease in unmet need. Women of the 15–19 year age group were about 3 times more likely to have an unmet need than women of the age group (20–24 years) (Table 3).

**Table 3:** Biosocial factors and Unmet need by Young Married Women in slums, UP, India

BIOSOCIAL CHARACTERISTICS	TOTAL WOMEN (***n*** = 362) (%)	UNMET NEED		CRUDE ODDS RATIO (95%CI)	ADJUSTED ODDS RATIO (95%CI)
		YES (n = 296) (%)	NO (***n*** = 66) (%)		
**Age of the women (Years)**					
15–19 (Reference)	99 [27.3]	90 (90.9) [30.4]	9 (9.1) [13.6]		
20–24	263 [72.7]	206 (78.3) [69.6]	57 (21.7) [86.4]	0.361 (0.172–0.762)**	0.341 (0.121–0.958)*
**Religion of the women** [8]					
Hindu	319 [88.1]	266 (83.4) [89.9]	53 (16.6) [80.3]	2.175 (1.064–4.443)*	4.369 (1.193–15.994)*
Muslim (Reference)	43 [11.9]	30 (69.8) [10.1]	13 (30.2) [19.7]		
**Caste / Tribe of the women** [8]					
Scheduled Caste / Scheduled Tribe (SC/ST) (Reference)	129 [35.6]	113 (87.6) [38.2]	16 (12.4) [24.2]		
Others	233 [64.4]	183 (78.5) [61.8]	50 (21.5) [75.8]	0.518 (0.282–0.954)*	–
**Level of Education of the women** [8]					
Illiterate (Reference)	70 [19.3]	65 (92.9) [22.0]	5 (7.1) [7.6]		
Literate	292 [80.7]	231 (79.1) [78.0]	61 (20.9) [92.4]	0.291 (0.112–0.755)*	0.124 (0.029–0.539)**
**Husband’s Level of Education** [8]					
Illiterate (Reference)	85 [23.5]	76 (89.4) [25.7]	9 (10.6) [13.6]		
Literate	277 [76.5]	220 (79.4) [74.3]	57 (20.6) [86.4]	0.457 (0.216–0.967)*	–
**Employment Status of the Women**					
Unemployed (Reference)	298 [82.3]	240 (80.5) [81.1]	58 (19.5) [87.9]		
Employed	64 [17.7]	56 (87.5) [18.9]	8 (12.5) [12.1]	1.692 (0.764–3.744)	–
**Type of family** [8]					
Nuclear (Reference)	222 [61.3]	183 (82.4) [61.8]	39 (17.6) [59.1]		
Joint	140 [38.7]	113 (80.7) [38.2]	27 (19.3) [40.9]	0.892 (0.518–1.536)	–
**Socio-economic Status (Based on modified Kuppuswamy Scale - 2016)** [22]					
Lower and Upper lower (Reference)	273 [75.4]	230 (84.2) [77.7]	43 (15.8) [65.2]		
Middle and above	89 [24.6]	66 (74.2) [22.3]	23 (25.8) [34.8]	0.536 (0.302–0.954)*	–
**Duration of stay in the slum**					
< 1 year (Reference)	109 [30.1]	97 (89.0) [32.8]	12 (11.0) [18.2]		
≥ 1 year	253 [69.9]	199 (78.7) [67.2]	54 (21.3) [81.8]	0.456 (0.233–0.892)*	-

(Row %) [Column%]*p < 0.05, **p < 0.005, ***p < 0.001

##### Religion and caste:

Religion was found to be significantly associated with unmet need for family planning services. More unmet need was observed among Hindus (83.4%) as compared to Muslims (69.8%) (COR: 2.17, CI: 1.06–4.44) (Table 3).

Majority (87.6%) of the women of scheduled caste / scheduled tribe (SC/ST) category had an unmet need for family planning services. Women belonging to the other categories were significantly less likely to have an unmet need than those belonging to SC / ST category (COR: 0.52, CI: 0.28–0.95) (Table 3).

##### Level of education:

The unmet need of family planning services was found to be significantly higher among the illiterate women (92.9%). Women who were literate were less likely of having unmet need for family planning services as compared to illiterate women (COR:0.29, CI: 0.11–0.75). Also women whose husband was educated were less likely of having unmet need as compared to those having uneducated husband (COR: 0.46, CI: 0.21–0.96) (Table 3).

##### Socioeconomic and employment status:

Unmet need was high (81.1%) among the unemployed women. It was only 18.9% in the employed women. No statistically significant association was observed between working status of women and unmet need for family planning. Unmet need for family planning services was also found to be high (84.2%) among women from lower and upper lower socio-economic class in comparison to the women belonging to middle and upper middle class (74.2%) (Table 3).

##### Duration of stay in the slum:

Women who were residing for more than a year in the slums were less likely (78.7%) to have an unmet need than those residing in the slum for less than a year (89.0%) and the association was statistically significant (COR:0.46, CI: 0.23–0.89) (Table 3).

#### Fertility related factors

##### Duration of marriage:

Women who were married for less than 1 year were significantly more likely to have an unmet need (92.8%; COR: 0.28, CI: 0.11–0.68), in comparison to women who were married for more than a year (Table 4).

**Table 4:** Fertility related factors and Unmet need by Young Married Women in slums, UP, India

CHARACTERISTICS	TOTAL WOMEN (n = 362) (%)	UNMET NEED		CRUDE ODDS RATIO (95%CI)	ADJUSTED ODDS RATIO (95%CI)
		YES (***n*** = 296) (%)	NO (***n*** = 66) (%)		
**Duration of marriage**					
< 1 year (Reference)	83 [22.9]	77 (92.8) [26.0]	6 (7.2) [9.1]		
≥ 1 year	279 [77.1]	219 (78.5) [74.0]	60 (21.5) [90.9]	0.284 (0.118–0.685)*	0.045 (0.008–0.244)***
**Total number of pregnancies**					
0 (Reference)	125 [34.5]	91 (72.8) [30.7]	34 (27.2) [51.5]		
≥ 1	237 [65.5]	205 (86.5) [69.3]	32 (13.5) [48.5]	2.217 (1.290–3.811)**	10.909 (3.854–30.877)***
**Total no of living children (*n*** **= 237)**					
0 (Reference)	11 [4.7]	5 (45.5) [2.5]	6 (54.5) [18.2]		
≥ 1	226 [95.3]	199 (88.0) [97.5]	27(12.0) [81.8]	8.844 (2.526–30.964)**	**–**
**Total no of male children (*n*** **= 226)**					
0 (Reference)	56 [24.8]	48 (85.7) [24.1]	8 (14.3) [29.6]		
≥ 1	170 [75.2]	151 (88.8) [75.9]	19 (11.2) [70.4]	1.325 (0.545–3.218)	–
**Desired number of children**					
< 2 (Reference)	181 [50.0]	159 (87.8) [53.7]	22 (12.2) [33.3]		
≥ 2	181 [50.0]	137 (75.7) [46.3]	44 (24.3) [66.7]	0.431 (0.246–0.755)**	0.165 (0.059–0.458)**

(Row %) [Column%]*p < 0.05, **p < 0.005, ***p < 0.001

##### Total number of pregnancies, number of living children, number of male children and desired number of children:

Parous women were 2.22 times more likely to have an unmet need than nulliparous women and this association was found to be statistically significant (Table 4). Women who had one or more living children had a high (88.0%) unmet need for family planning services and majority (88.8%) of the women with a male child had an unmet need for family planning services. Women who had one or more living children were 8.84 times more likely to have an unmet need than women with no living children and this association was found to be statistically significant. The association between number of male children and unmet need was found to be statistically insignificant (Table 4). The number of children desired by the women was found to have a statistically significant association with unmet need for family planning services; with higher unmet need (87.8%) in women desiring < 2 children. Women who desired ≥2 children were significantly less likely to have an unmet need than women desiring < 2 children (COR: 0.43, CI: 0.24–0.75) (Table 4).

##### Knowledge of contraceptive methods and its access:

Women who did not have any knowledge of contraceptive methods had statistically significant (COR: 0.36, CI: 0.18–0.71**)** high unmet need (90.3%) for family planning services. Majority (83.1%) of the women who did not have any knowledge of place where family planning services are available near their slum had an unmet need. Women who had knowledge of availability of family planning services at the U-PHC were significantly less likely to have an unmet need than women with no knowledge (COR: 0.35, CI: 0.13–0.94) (Table 5).

**Table 5:** Knowledge of family planning methods and services, Contact with Health worker, Media Exposure and Unmet need by Young Married Women in slums, UP, India

CHARACTERISTICS	TOTAL WOMEN (n = 362) (%)	UNMET NEED		CRUDE ODDS RATIO (95%CI)	ADJUSTED ODDS RATIO (95%CI)
		YES (n = 296) (%)	NO (n = 66) (%)		
**Knowledge of contraceptive methods**					
Yes	238 [65.7]	184 (77.3) [62.2]	54 (22.7) [81.8]	0.365 (0.187–0.712)**	0.273 (0.102–0.731)*
No (Reference)	124 [34.2]	112 (90.3) [37.8]	12 (9.7) [18.2]		
**Knowledge of availability of Family Planning services at the U- PHC**					
Yes	19 [15.3]	12 (63.2) [4.1]	7 (36.8) [10.6]	0.356 (0.135–0.943)*	–
No (Reference)	343 [94.7]	284 (82.8) [95.9]	59 (17.2) [89.4]		
**Media Exposure of message on Family planning on TV or Radio in the last 6 months**					
Yes	146 [40.3]	119 (81.5) [40.2]	27 (18.5) [40.9]	0.971 (0.564–1.671)	**–**
No (Reference)	216 [59.7]	177 (81.9) [59.8]	39 (18.1) [59.1]		
**Contact with ANM**					
Yes	50 [13.8]	33 (66.0) [11.1]	17 (34.0) [25.8]	0.362 (0.187–0.700)**	0.377 (0.148–0.963)*
No (Reference)	312 [86.2]	263 (84.3) [88.9]	49 (15.7) [74.2]		

(Row %) [Column%]*p < 0.05, **p < 0.005, ***p < 0.001

##### Media exposure:

Less than half (40.3%) of the young married women were exposed to family planning message on TV/ radio. The association between media exposure and unmet need for family planning services was found to be statistically insignificant (Table 5).

##### Contact with health worker:

Association between contact of ANM (Auxiliary Nurse Midwife) during household visits in the slums or during HNDs (Health and Nutrition Days) and unmet need for family planning services was found to be statistically significant. Women who did not have a contact with ANM were about 3 times more likely to have an unmet need than women who had a contact (Table 5).

##### Autonomy status of women:

Women who had “no autonomy” in their family had a higher (89.2%) unmet need for family planning services. Women who had “some autonomy” and those who had “autonomy” were less likely to have an unmet need than women with “no autonomy” (COR: 0.46, CI: 0.24–0.87 & COR: 0.25, CI: 0.10–0.58 respectively) (Table 6).

**Table 6:** Decision making authority of woman, Husband and Family member’s attitude towards family planning and Unmet need by Young Married Women in slums, UP, India

CHARACTERISTICS	TOTAL WOMEN (n = 362) (%)	UNMET NEED		CRUDE ODDS RATIO(95%CI)	ADJUSTED ODDS RATIO(95%CI)
		YES (n = 296) (%)	NO (n = 66) (%)		
**Autonomy Status** [23]					
Has autonomy	40 [11.1]	27 (67.5) [9.1]	13 (32.5) [19.7]	0.251 (0.107–0.589)**	0.276 (0.084–0.909)*
Has some autonomy	183 [50.5]	145 (79.2) [49.0]	38 (20.8) [57.6]	0.462 (0.242–0.879)*	0.454 (0.191–1.082)
Has no autonomy (Reference)	139 [38.4]	124 (89.2) [41.9]	15 (10.8) [22.7]		
**Husband’s Attitude towards Family Planning**					
Favourable	58 [16.1]	41 (70.7) [13.9]	17 (29.3) [25.8]	0.417 (0.221–0.787)*	0.299 (0.104–0.861)*
Unfavourable (Reference)	304 [83.9]	255 (83.9) [86.1]	49 (16.1) [74.2]		
**Discussed family planning with anyone**					
Yes (Reference)	117 [32.3]	98 (83.8) [33.1]	19 (16.2) [28.8]		
No	245 [67.7]	198 (80.8) [66.9]	47 (19.2) [71.2]	0.817 (0.455–1.466)	–
**Motivated for adoption of family planning methods**	52 [14.4]	41 (78.8) [13.9]	11 (21.2) [16.7]	0.804 (0.389–1.662)	–
Motivated by Husband/ Other family members / Friends / Relatives	43 [11.9]	29 (67.4) [9.8]	14 (32.6) [21.2]	0.481 (0.225–1.032)	–
Motivated by Health care provider	32 [8.8]	25 (78.1) [8.4]	7 (21.9) [10.6]	0.893 (0.225–3.547)	–
**Opposition to Contraceptive use by anyone**					
Yes	42 [11.6]	40 (95.2) [13.5]	2 (4.8) [3.0]	5.000 (1.177–21.237)*	7.358 (1.327–40.798)*
No (Reference)	320 [88.4]	256 (80.0) [86.5]	64 (20.0) [97.0]		

(Row %) [Column%]*p < 0.05, **p < 0.005, ***p < 0.001

##### Motivation and opposition to contraceptive use:

Women whose husbands had an unfavorable attitude towards family planning had a high (83.9%) unmet need. Women with husbands having a favorable attitude were found to be less likely to have an unmet need as compared to women whose husband had an unfavorable attitude (COR: 0.42, CI: 0.22–0.78) (Table 6). Also the unmet need was found to be more (66.9%) in absence of any discussion of family planning with husband and with others; the association being statistically insignificant. On the other hand only 13.9% of the women who were motivated to use contraceptive methods had an unmet need. Unmet need was found to be less in those women who were motivated to use family planning methods by husbands, by other family members / friends / relatives, by health care providers but this association was found to be statistically insignificant (Table 6). About 11.6% young married women reported opposition to contraceptive use. Women having opposition to contraceptive use were 5.00 times more likely to have an unmet need than women with no opposition and this association was found to be statistically significant (Table 6).

### Multivariate logistic regression

Factors found to be statistically significant (*p* value < 0.05) in bivariate analysis were subjected to conditional multiple logistic regression for adjustment and controlling the effect of confounding variables. Several factors that were found to be statistically significant on bivariate analysis lost their significant on multivariate analysis which could be partly explained due to co-linearity and possible confounding observed between predictor variable. Age of the women, educational status of the women, duration of marriage, number of pregnancies, knowledge of contraceptive methods, opposition to contraceptive use and contact with ANM showed independently significant association with unmet need for family planning. Women of 20–24 year age group were significantly less likely to have an unmet need than women of the lower age group (AOR: 0.34, CI: 0.12–0.95). Women who were literate were significantly less likely to have an unmet need for family planning as compared to illiterate women (AOR: 0.12, CI: 0.02–0.53) (Table 3). Parous women were significantly more likely to have an unmet need than nulliparous women (AOR: 10.90, CI: 3.8–30.8) (Table 4). Women who had any knowledge of contraceptive methods were significantly less likely to have an unmet need than women with no knowledge (AOR: 0.27, CI: 0.10–0.73) (Table 5). Women having opposition to contraceptive use were significantly more likely to have an unmet need than women with no opposition (AOR: 7.36, CI: 1.3–40.7) (Table 6). Women that had a contact with ANM were significantly less likely to have an unmet need than women who did not have a contact (AOR: 0.38, CI: 0.14–0.96) (Table 5).

## Discussion

More than half (55.3%) of the young married women living in the slums were having an unmet need for family planning, of which 40.9% was for spacing and 14.4% for limiting. Almost all (95.6%) women in the younger age group (15-19 years) had an unmet need for spacing methods as compared to older age group (64.6%). This is much higher than the unmet need for family planning as reported by NFHS-IV [6] in Uttar Pradesh (13.4%) and in Lucknow (14.5%). The unmet need in YMW is even higher than that reported in rural Uttar Pradesh (19.6%) [6]. Shukla, M., et. al., [11] in urban slums of Lucknow also found a higher unmet need (62.5%) among young married women. However, Pal, A., et. al., [10] reported very high (85.5%) unmet need in the urban slums of Lucknow about a decade ago. In selected slums unmet need is higher than that found by Sherin, R., et. al., [24] (23.4%) in their study in Rajasthan.

Age of the women was found to be a significant predictor for unmet need of family planning. In the present study the unmet need for family planning was found to be significantly higher in the age group of 15–19 years (90.9%). Women of the younger age group (15–19 years) are more likely to have an unmet need as women of the age group 20–24 years are more educated and have more knowledge and experience of contraception [25]. They tend to be more mature and play a role in decision making, thereby less prone to have an unmet need [25]. Similar to these findings the younger women in the present study are reported to have significantly less knowledge and poor access to information, lack of decision making power, are shy / hesitant and are undermined by socio-cultural expectations of early marriage and childbearing. Duration of marriage less than 1 year was found as one of the determinants of unmet need in the study. Begum, S., *et. al.,* [26] perceived that this high unmet need among newly-wed couples might be due to socio-cultural practice in the Bangladeshi community to have a child immediately after marriage. Socio cultural practices of Indian community are more or less similar to the Bangladeshi community. Begum, S., *et. al.,* [26] also reported that sometimes the health providers impose barriers in accessing FP services by young women and, resulting in increase in unmet need for services among this group .

Education level of the women emerged out as one of the strong predictor for unmet need for FP services in the urban slums. Majority (92.9%) of the women in the present study with lower level of education were found to have an unmet need for family planning services and unmet need was found to decrease with increase in level of education with only 79.1% among those who were literate having an unmet need for family planning services. Similar findings were reported by Sherin, R., [24], Wulifan., *et. al.,* [27] and Hamsa, L., *et. al.,* [28] who also observed that a lower level of education was significantly associated with higher unmet need.

In our study, majority of both the multiparous (86.5%) and nulliparous (72.8%) women expressed no desire for childbirth at present but were still not using any of the contraceptive methods. Almost all of the nulliparous and primiparous women had an unmet need for spacing whereas two-third of the multiparous women (61.8%) had an unmet need for limiting methods. Unmet need for family planning services was significantly higher in women with more number of pregnancies but nulliparous women also constituted the major bulk of those having an unmet need. This is in accordance to the findings of studies done in developing and developed nations around the world [24, 27, 28], which reported a lower unmet need among nulliparous women. Contrary to this Imasiku., *et. al.,* [29] and Shukla, M., *et. al.,* [11] found unmet need to be more in nulliparous women. Calhoun, LM., *et. al.,* [30] found that providers restrict clients’ access to spacing and long-acting and permanent methods of family planning based on parity. Similar views were echoed by Begum, S., *et. al.,* [26].

Unmet need was also found to be significantly associated with the number of children that are desired by a woman, which is in concurrence with study done by Bhattathiry, MM., and Ethirajan, N., [31].

In concurrence with Mosha, I., [32] and Woldemicael, G., and Beaujot, R., [33] who found that women who had less autonomy in the family; were more likely to have an unmet need. Significant association was found between the autonomy of the young married women and unmet need. The study reported that most of the women in the younger age group had no autonomy in the family and hence more prone to unmet need.

Chafo, K., [21] attributed the availability of an enabling environment in the family helpful for women in implementing fertility desires and fulfilling their contraceptive needs. In this study also significant association was found between husband’s favorable attitude for family planning and low unmet need. However, only 16.1% reported that their husbands were favorable towards family planning methods. This is similar to the findings of other studies done in various low and middle income countries among slum women aged 15–24 years [10, 31, 34, 35]. In accordance to other researchers (Kabagenyi, A*., et. al.,* [36] and Hall, MAK., *et. al.,* [37], the present study also found significantly high unmet need among young women who faced opposition to contraceptive use by the husband or families. In this study 45% of YMW reported opposition from either husband or other family members. This needs to be dealt by utmost attention by the program managers during planning for FP services for this group in the slums. A study of reproductive health service providers in urban Uttar Pradesh highlighted that providers also imposed restrictions to younger clients’ access to FP methods based on partner consent [30]. Approximately one quarter of midwives restricted client access to pills and condoms based on partner consent and nearly 75% restricted access to the IUCD based on partner consent [30]. The pattern that has emerged from the study that a particular profile of clients-under educated, poor, having few or no children, not having the support of their partner, and newly-wed women; are less likely to receive FP counseling by a provider in the urban Uttar Pradesh [30].

Similar to that reported by other studies [28, 31, 33], who found that women were less likely to have unmet need if they were aware of contaceptive methods and site from where FP can be procured, our study also observed significantly high unmet need among women who have low knowledge of contraceptive methods and place for FP services procurement. In the present study knowledge of contraceptive methods was found low (33.9%) among the young women (15–19 years) and 76.7% were not aware of place from where they can avail the FP services.

Role of frontline workers is crucial in uptake of family planning services by the community. Researchers in various parts of the world [21, 35, 38, 39] found the met need of family planning to be significantly higher among those women who had a contact with ANM. Similar findings are reported in the present study where unmet need for family planning services was found to be significantly higher among those women who had no contact with ANM (89.1%) as compared to those who had a contact with ANM (11.1%). In this study only 21.3% women had any contact with the ANM and about 8.8% women were recommended by health care provider for adoption of FP methods. Contact with health worker was almost negligible in the case of 15–19 years age group. Wulifan., *et. al.,* [27] stated that though women of reproductive age in low and middle income countries are in favor of birth spacing but they were less likely to engage in family planning discussion with health workers in comparison to the older women. This reluctance in actively expressing their FP needs is in parts explained by prevailing stigma, shyness, hesitation, embarrassment, myths / misconceptions and socio-cultural expectations attached to contraceptive use in young as found in the present study. It reflects the dire need for the national and regional program managers to take into consideration the favorable effect of contact with ANMs as a golden opportunity to increase the use of family planning methods especially by young married women living in urban slums. Recently Urban-ASHA has been deployed under the National Urban Health Mission [17] and it is expected that they will reduce the unmet need in these urban slums.

The main reasons for unmet need for family planning services among the young married women in the present study were found to be shyness / embarrassment / hesitancy followed by lack of knowledge regarding family planning method as well as their accessibility. About 40% of the women had a negligent attitude towards family planning. Opposition for contraceptive use was faced by one third of the women. In concurrence to the resent study, Sultana, B., *et. al.,* [40] in their study in urban slums of Pondicherry, also found that client related factors (lack of knowledge, shyness, etc.); and contraception related factors (availability, accessibility, affordability, side effects) were the cause for unmet need. Huda, FA., *et. al.,* [41] in the study among married adolescent girls in slums of Bangladesh, reported that lack of knowledge of the available methods, family pressure to prove fertility, opposition from husbands and mothers-in-laws were the main reasons for unmet need. Nazish, R., *et. al.,* [42] in their study in Uttar Pradesh found that the major reasons for unmet need for FP were opposition from husband or family, poor accessibility of the method and negligent attitude of the women towards family planning.

The coverage of a large slum population and use of a strong methodology enhances the internal and external validity of the research work. However considering the important role men play in the dynamics of family planning, their non inclusion in the present study may not reflect the overall perspective of the couple with regard to the use of family planning services. Therefore further studies can be done for in-depth exploration of these factors.

## Conclusions

Unmet need for family planning was found to be very high among the young married women of urban slums. The study identifies the focus areas which have to be addressed to achieve reduction in unmet need and there by attainment of the desired goal of population stabilization and better reproductive and maternal health.

Molding the minds of young generation at an early stage by inculcating reproductive and sexual education as a part of routine school health services could go a long way in motivating them to adopt contraceptive use in future and subsequently follow a healthy fertility behavior. Formation of community based peer system will provide an opportunity for holistic discussion about family planning methods. These community based peer groups will help the young women to overcome embarrassment, shyness, or hesitation and will also give them autonomy to avail FP services. A comprehensive approach should be used by the health worker working in these slums to provide counseling services not only to the young married woman but to all stakeholders. Training should be imparted to health workers to improve their interpersonal behavior change communication skills to tackle the myths, misconceptions, embarrassment / hesitancy / shyness and fears regarding contraceptive use among this young population. Apart from training it is also importance to sensitize the health workers that within this age group there are various vulnerable sub sections with their diverse need for FP services; newlywed, recently settled in urban part, nulliparous, less educated, woman with no autonomy and with opposition from partners and families, warranting a combined and coordinated approach directed towards each subgroup.

## Supplementary information

**Additional file 1.** Final questionnaire. Questionnaire.

## Data Availability

The datasets generated and / or analyzed during the current study are not publicly available due to the topic of the study and concerns regarding confidentiality of the data but are available from the corresponding author on reasonable request.

## Abbreviations

YMW: Young Married Woman
FP: Family Planning
UP: Uttar Pradesh
UPHC: Urban Primary Health Centre
ANM: Auxiliary Nurse and Midwife
VHND: Village Health and Nutrition Day
ASMFR: Age Specific Marital Fertility Rate
CPR: Contraceptive Prevalence Rate
VHND: Village Health and Nutrition Days
BMC: Bal Mahila Chikitsalays
SC/ST: Scheduled Caste/ Scheduled Tribe
OBCs: Other Backward Classes
COR: Crude Odds Ratio
AOR: Adjusted Odds Ratio
