# Correction to: Unmet need for family planning services among young married women (15–24 years) living in urban slums of India

**DOI:** 10.1186/s12905-020-01076-5

**Published:** 2020-09-24

**Authors:** Kriti Yadav, Monika Agarwal, Mukesh Shukla, Jai Vir Singh, Vijay Kumar Singh

**Affiliations:** 1 Department of Community & Family Medicine, All India Institute of Medical Sciences, Bhopal, MP India; 2 Department of Community Medicine & Public Health, K.G. Medical University, Lucknow, UP India; 3 Vir Chandra Singh Garhwali Government Institute of Medical Science and Research, Srinagar, Pauri Garhwal, Uttarakhand India; 4 Hind Institute of Medical Sciences, Lucknow, UP India

**Correction to: BMC Womens Health (2020) 21:46**

**https://doi.org/10.1186/s12905-020-01010-9**

Following publication of the original article [1], we were notified of an error in the affiliation of the first author Kriti Yadav:

Incorrect affiliation:

- Assistant Professor, Vir Chandra Singh Garhwali Government Institute of Medical Science and Research, Srinagar, Pauri Garhwal, Uttarakhand, India

Correct affiliation:

- MD, Senior Resident, Department of Community & Family Medicine, All India Institute of Medical Sciences, Bhopal, MP, India.
