# Supplementary material for: Unmet need for family planning services among young married women (15–24 years) living in urban slums of India
Source: BMC Womens Health. 2020 Sep 3;20:187. doi: 10.1186/s12905-020-01010-9 (PMC7469334; doi:10.1186/s12905-020-01010-9)
Supplement: Supplementary file 1 — Additional file 1. Final questionnaire. Questionnaire. [file 12905_2020_1010_MOESM1_ESM.docx]

**DEPARTMENT OF COMMUNITY MEDICINE AND PUBLIC HEALTH**

**KING GEORGE’S MEDICAL UNIVERSITY, LUCKNOW**

**UTILIZATION OF FAMILY PLANNING SERVICES BY YOUNG MARRIED WOMEN LIVING IN URBAN SLUMS OF LUCKNOW**

FORM No. Date: Starting Time: End Time:

Nagar Nigam Zone:

Name of the Slum:

Name of the designated U-PHC:

Distance of designated U-PHC from Slum: Kms

| **S No.** | **GENERAL INFORMATION OF THE RESPONDENT** | |
| --- | --- | --- |
|  | Name of the respondent |  |
|  | Age in completed years |  |
|  | Duration of stay in the city ( In months/years) |  |
|  | Religion | 1. Hindu 2. Muslim 3. Christian 4. Sikh 5. Others (specify) |
|  | Caste | 1. General 2. OBC 3. SC/ST |
|  | Education of the respondent | A B C D E F G |
|  | Occupation of the respondent | A B C D E F G |
|  | Husband’s education | A B C D E F G |
|  | Husband’s occupation | A B C D E F G |
|  | Type of family | 1. Nuclear family 2. Joint family 3. Three-Generation family |
|  | Family Income (Rs. Per Month) |  |
|  | Socioeconomic status of Kuppuswamy (Based on Education and Occupation of Head of the Household and Family Income) | 1. Lower (I) 2. Upper Lower (II) 3. Middle (III) 4. Upper Middle (IV) 5. Upper (V) |
|  | 1. Does this household have electricity? 2. Does the household own a bed? 3. Does the household own a chair/table? 4. Does the household own a mobile? 5. Does the household own cooking gas? 6. Does the household own a pressure cooker or pressure pan? 7. Does the household own an electric fan? 8. Does the household own a radio/television? 9. Does the household own a bicycle, scooter, or motorcycle? 10. Does the household own a refrigerator / cooler? 11. Does your house have water pump? | Yes / No  Yes / No  Yes / No  Yes / No  Yes / No  Yes / No  Yes / No  Yes / No  Yes / No  Yes / No  Yes / No |
|  | Socio Economic Scale | 1. Lower 2. Upper Lower 3. Lower Middle 4. Upper Middle 5. Upper |
|  | **MARITAL AND OBSTETRIC HISTORY OF THE RESPONDENT** | |
|  | Age of the respondent at marriage (In years) |  |
|  | Age of the respondent at gauna (In years) |  |
|  | Age of the respondent at the birth of first child (In years) |  |
|  | Total No. of pregnancies |  |
|  | Total No. of live births | Total : Male: Female: |
|  | Total No. of abortions (if any) |  |
|  | Total No. of children alive at present | Total : Male: Female: |
|  | Place of delivery of the last child | 1. Home 2. Government health facility 3. Private facility 4. Others (specify) |
|  | **KNOWLEDGE AND ATTITUDE OF THE RESPONDENT ABOUT FAMILY PLANNING** | |
|  | In your opinion, what is the ideal age for a girl to get married? | 1. At the onset of menarche 2. 14-16 years 3. 16-18 years 4. >18 years 5. Other specific answer |
|  | Which one is the ideal age for a woman to have first child? | 1. Just after marriage 2. 1-2 years after marriage 3. >2 years after marriage 4. Other specific answer |
|  | In your opinion what is the ideal number of children that a woman should have? | 1. One 2. Two 3. More than two   Male : Female: No Sex Differentiation |
|  | What is the ideal age space between two children? | 1. One year 2. One to two years 3. Two to three years 4. Three to five years 5. Five years or more 6. Don’t Know |
|  | When you started your family how many children did you and your husband want? | 1. Never Thought of it 2. One 3. Two 4. More than two   Male : Female: No Sex Differentiation |
|  | After your marriage when did you and your husband want your first child? | 1. Never Thought of it 2. Just after marriage 3. 1-2 years after marriage 4. >2 years after marriage 5. Others (specify) |
|  | Do you want any more children? (When ?) |  |
|  | Did you ever discuss about these matters (the number of children and spacing) with your husband? | Yes  No  (IF NO SKIP TO QUESTION No.32) |
|  | If yes, then what was your husband’s attitude? | A B C D E F  (SKIP TO QUESTION No.33) |
|  | If no, why not? | A B C D E F G H |
|  | Did you discuss about these matters (the number of children and spacing) with anyone else? | Yes  No  (IF NO SKIP TO QUESTION No.35) |
|  | If yes, with whom? | 1. Mother / Mother in law 2. Sister / Sister in law 3. Friend / Neighbour 4. Other (specify)   (SKIP TO QUESTION No.36) |
|  | If no, why not? | A B C D E F G H |
|  | Did anyone motivate you to use contraceptives? | Yes / No  (IF NO SKIP TO QUESTION No.38) |
|  | If yes, who? | 1. Husband 2. Mother / Mother in law 3. Sister / Sister in law 4. Friend / Neighbour 5. Health worker 6. Others (specify) |
|  | Do you know about JSY? | Yes / No |
|  | Do you know about any program run by the Government for family planning? | Yes / No |
|  | Is there any advantage of having a small family / family planning? |  |
|  | Is there any disadvantage of having a small family / family planning? |  |
|  | Did you get any education about family planning before you got married? | Yes  No |
|  | Which family planning methods do you know about? | 1. **MODERN METHODS:**   A B C D E F G H   1. **TRADITIONAL METHODS:**   A B   1. **Other (specify)** 2. **Don’t know any method**   (IF DONT KNOW ANY METHOD SKIP TO QUESTION No.46) |
|  | From where did you come to know about these methods? | 1. **Media Sources:**   A B C D E F G   1. **Health Sources:**   A B C D E F G H   1. **Community Sources:**   A B C D   1. **Interpersonal Sources:**   A B C D E F   1. **Others (Specify)** |
|  | Have you heard/seen any message on family planning on radio/TV in the last six months? | Yes  No |
|  | Do you know of any incentive which is given for family planning by the government? | Yes  No |
|  | Do you know any place where family planning services are provided free of cost? | Yes  No  (IF NO SKIP TO QUESTION No.47) |
|  | If yes, which place? | 1. **Public Health Sector:**   A B C D E F G H I   1. **Medical college hospitals** 2. **NGO or trust hospital/clinic** |
|  | **RESPONDENT’S AUTONOMY IN THE FAMILY** | |
|  | In your family who decides how the money you earn will be used? | A B C D |
|  | In your family who usually makes decisions about health care for yourself? | A B C D |
|  | In your family who usually makes decisions about making major household purchase? | A B C D |
|  | Are you usually allowed to go to the following places alone, only with someone else or not at all?   1. To the market 2. To the health facility 3. To family/relatives house 4. To places outside this community | Alone/Only with someone else/Not at all  Alone/Only with someone else/Not at all  Alone/Only with someone else/Not at all  Alone/Only with someone else/Not at all |
|  | **USE OF CONTRACEPTIVE BY THE RESPONDENT** | |
|  | At present are you planning to get pregnant? | Yes  No  (IF YES SKIP TO QUESTION No.68) |
|  | Are you currently using any of the contraceptive methods? | Yes  No  (IF NO SKIP TO QUESTION No.68) |
|  | **FOR CONTRACEPTIVE CURRENT USERS** | |
|  | What method are you using? | 1. **MODERN METHODS:**   A B C D E F G H   1. **TRADITIONAL METHODS:**   A B   1. **Other (specify)**   (IF NOT b) THEN SKIP TO QUESTION No. 57) |
|  | Why are you not using any Modern Method of contraception? | (SKIP TO QUESTION No. 68) |
|  | Duration of use | (in months/years) |
|  | Do you use it regularly? | Yes / No  (IF YES THEN SKIP TO QUESTION No. 62) |
|  | If No, Why do you use it irregularly? |  |
|  | Do you use any other method(s) when you are not using this method? | Yes / No  (IF NO THEN SKIP TO QUESTION No.62) |
|  | If Yes, Which method(s)? | 1. **MODERN METHODS:**   A B C D E F G H   1. **Other (specify)** |
|  | Are you satisfied with the method you are currently using? | Yes  No |
|  | Are you facing any problems regarding the usage of this method? | Yes  No  (IF NO SKIP TO QUESTION No.65) |
|  | If yes, what? |  |
|  | Have you used any other contraceptive before using the current one in the last one year? | Yes / No  (IF NO SKIP TO QUESTION No.73) |
|  | If yes, Which method(s)? | 1. **MODERN METHODS:**   A B C D E F G H   1. **Other (specify)** |
|  | Why did you discontinue the method? | A B C D E F G H I J K L M N O P Q R S T U  (SKIP TO QUESTION No.73) |
|  | Have you ever used any of the contraceptive methods? | Yes  No  (IF NO SKIP TO QUESTION No.82) |
|  | **FOR CONTRACEPTIVE EVER USERS** | |
|  | When was the last time you used a method? | 1. Within 3 months 2. More than 3-6 months ago 3. More than 6 months -1 year ago 4. More than 1 year ago |
|  | What was the method? | 1. **MODERN METHODS:**   A B C D E F G H   1. **Other (specify)** |
|  | Duration of use during last time | (in months) |
|  | Why did you stop using the method? | A B C D E F G H I J K L M N O P Q R S T U |
|  | **FOR BOTH CURRENT AND EVER CONTRACEPTIVE USERS** | |
|  | Between you and your husband who decided to use this method? | 1. Self 2. Husband 3. Both 4. Others ( Specify ) |
|  | Did anyone oppose your decision of using contraceptives? | Yes  No  (IF NO SKIP TO QUESTION No.77) |
|  | If yes, who? | 1. Husband 2. Mother in law 3. Father in law 4. Sister in law 5. Whole Family 6. Other (specify) |
|  | If yes, why? |  |
|  | Did you suffer from any of the side-effects of this method? | Yes  No  (IF NO SKIP TO QUESTION No.80) |
|  | Did you seek any treatment for these side-effects? | Yes  No  (IF YES SKIP TO QUESTION No.80) |
|  | If no, then why not? | A B C D E F G H I J |
|  | From where did you procure this family planning method? | 1. **Public Health Sector:**   A B C D E F G H I J   1. **Medical college hospitals** 2. **NGO or trust hospital/clinic** 3. **Private health sector :**   A B C D E F G   1. **Other source:**   A B   1. **Other (specify)**   (IF NOT U-PHC THEN 81 ELSE SKIP TO QUESTION No.83) |
|  | Did you face any problem in procuring the method? | (SKIP TO QUESTION No.83) |
|  | **FOR CONTRACEPTIVE NON-USERS** | |
|  | Why haven’t you ever used any contraceptive method? | 1. **Fertility Related Reasons:**   A B C D E F G H   1. **Opposition To Use:**   A B C D   1. **Lack of Knowledge:**   A B C D   1. **Method-Related Reasons:**   A B C D E F G   1. **Fatalistic:**   A   1. **Health facility related reasons:**   A B C D E F   1. **Other :**   A B   1. **Don't Know** |
|  | Do you think you will use/continue to use any contraceptive method to delay or avoid getting pregnant at any time in the future? | Yes  No  Don’t Know  (IF YES SKIP TO QUESTION No.85) |
|  | If no, why not? | A B C D E F G H I J K L M N O P Q R S |
|  | **EMERGENCY CONTRACEPTIVE USAGE AND ABORTION RELATED INFORMATION** | |
|  | Do you know about emergency contraceptives? | Yes  No  (IF NO SKIP TO QUESTION No.88) |
|  | In the last 12 months have you ever used emergency contraceptive? | Yes  No  (IF NO SKIP TO QUESTION No.88) |
|  | If yes, how many times? |  |
|  | Have you ever done requesting abortion? | Yes  No  (IF NO SKIP TO QUESTION No.90) |
|  | If yes, what was the reason for requesting abortion? | 1. Pregnancy not planned 2. Too young to have first child 3. Too little birth space between children 4. Social/Economic reason |
|  | **UTILIZATION OF HEALTH SERVICES BY THE RESPONDENT** | |
|  | Which is the nearest government health facility? | 1. Medical college hospitals 2. Govt./Municipal hospital 3. Govt. Dispensary 4. UHC/UHP/UFWC 5. Don’t know   (IF iv. IS SELECTED THEN SKIP TO QUESTION No. 92) |
|  | Do you know about the U-PHC in your locality? | Yes/ No  (IF NO SKIP TO QUESTION No.124) |
|  | What health services are provided by this facility? | A B C D E F G H |
|  | Which services do you utilize from this facility? | A B C D E F G H  (IF NONE IS SELECTED THEN SKIP TO QUESTION No.105) |
|  | **FOR U-PHC UTILIZERS** | |
|  | How long does it take to go to the U-PHC? | Time in minutes( ) |
|  | What is the main mode of transport that is used to go there? | A B C D E F G H |
|  | Do you face problems in   1. Getting permission to go 2. Finding someone to go with 3. Having to take transport | Yes/No  Yes/No  Yes/No |
|  | Are the working hours of the facility convenient for you? | Yes  No |
|  | How long do you have to wait for consulting the staff/provider after arriving at the facility? | A B C D E F G |
|  | Do you feel that your waiting time is reasonable or too long? | 1. No waiting time; was seen immediately 2. Reasonable amount of time 3. Too long 4. Don’t know |
|  | During your visit, how were you treated by the  provider? | 1. Very well 2. Well 3. Not very well/Poorly |
|  | Do you have any concern regarding availability of a female healthcare provider? | Yes  No |
|  | Is there any problem related to the health facility? | Yes  No  (IF NO SKIP TO QUESTION No.104) |
|  | If yes, then what? |  |
|  | Do you utilize family planning services from the U-PHC? | Yes/ No  (IF YES SKIP TO QUESTION No.106) |
|  | Why are you not utilizing the family planning services provided by this facility? | A B C D E F G H I J K L M N O P Q R S T U  (SKIP TO QUESTION No.124) |
|  | **FOR U-PHC FAMILY PLANNING SERVICE UTILIZERS** | |
|  | Did the provider inform you about different family planning methods to delay or avoid getting pregnant? | Yes  No |
|  | Did the provider help you in selecting a family planning method? | Yes  No |
|  | Did the provider tell you about the possible side effects or problems you might have with this method? | Yes  No |
|  | Did the provider counsel you regarding how to solve these problems? | Yes  No |
|  | Did the provider show you any printed materials  on family planning during their discussion with  you? | Yes  No |
|  | Is your privacy/confidentiality maintained during the consultation? | Yes  No |
|  | Do you feel comfortable in asking questions during  your visits? | Yes  No |
|  | Are the desired services available as and when required? | Yes  No |
|  | Are the desired contraceptive available as and when required? | Yes  No |
|  | Were you given any printed materials on family  planning to take away with you during your visit? | Yes  No |
|  | Are the services costly? | Yes  No |
|  | Is the quality of services poor? | Yes  No |
|  | Are there any referral related issues? | Yes  No |
|  | Whether any incentives were provided for accepting any of the family planning services or not? | Yes  No |
|  | Are you satisfied with the services provided by this facility? | Yes  No  (IF YES SKIP TO QUESTION No.122) |
|  | If No, why? |  |
|  | Will you use this facility for health care services in  the future? | Yes  No |
|  | Will you recommend this facility to  Family/friends/neighbours? | Yes  No |
|  | **INFORMATION REGARDING ANM VISITS** | |
|  | Do you know that ANM is supposed to give you information/services regarding family planning? | Yes  No |
|  | Has there been any visit by the ANM in the last three months in your slum? | Yes  No  (IF NO SKIP TO QUESTION No.131) |
|  | During this visit what were the different services provided and matters talked about? | A B C D E F G H I J K L M N O P |
|  | Did she tell about any place from where you can avail family planning services? | Yes  No  (IF NO SKIP TO QUESTION No.129) |
|  | If yes, which facility did she tell about? | 1. Medical college hospitals 2. Govt./Municipal hospital 3. Govt. Dispensary 4. UHC |
|  | Are you satisfied by the family planning services provided by the ANM? | Yes  No  Not Applicable  (IF YES SKIP TO QUESTION No.131) |
|  | If no, then why not? |  |
|  | **HND/IMMUNIZATION DAY RELATED INFORMATION** | |
|  | Do you attend or did you attend any HND/Immunization day in the last three months? | Yes  No  (IF NO SKIP TO QUESTION No.135) |
|  | If yes, was family planning discussed during that session? | Yes  No  (IF NO SKIP TO QUESTION No.136) |
|  | Were you satisfied with the discussion? | Yes  No  (IF YES SKIP TO QUESTION No.136) |
|  | If no, why not? | (SKIP TO QUESTION No.136) |
|  | Why you don’t attend HND/Immunization day? | 1. Did not know 2. At a far place/ No transport 3. At a inconvenient time 4. No one to go with 5. No one at home 6. Busy with some work 7. Did not find it beneficial 8. Not Conducted |
|  | In your opinion do you think girl/woman should be educated regarding the use of contraceptives and family planning? | Yes  No  (IF NO THEN END INTERVIEW) |
|  | In your opinion what is the most appropriate time for imparting education regarding the use of contraceptives and family planning? |  |
|  | In your opinion which is the most ideal place / method for imparting education regarding the use of contraceptives and family planning? |  |
|  | In your opinion who is the most ideal person for imparting education regarding the use of contraceptives and family planning? |  |
|  | In your opinion, what should be done to improve family planning service delivery? |  |

| **DEPARTMENT OF COMMUNITY MEDICINE AND PUBLIC HEALTH**  **KING GEORGE’S MEDICAL UNIVERSITY, LUCKNOW**  **ASSESSMENT OF U-PHC FOR PROVISION OF FAMILY PLANNING SERVICES**  FORM No. Date: | | | | | | | | | | | | | | | | |
| --- | --- | --- | --- | --- | --- | --- | --- | --- | --- | --- | --- | --- | --- | --- | --- | --- |
| S No. | **GENERAL INFORMATION** | | | | | | | | | | | | | | | |
|  | Nagar Nigam Zone / Block: | | | | | | | | | | |  | | | | |
|  | U-PHC: | | | | | | | | | | |  | | | | |
|  | Name of the MO I / C: | | | | | | | | | | |  | | | | |
|  | Which year did this facility first begin offering health services / products? | | | | | | | | | | | Month:______  Year:_______ | | | | |
|  | How many days each week is the facility routinely open? | | | | | | | | | | | Number of days:______ | | | | |
|  | What are the opening hours? | | | | | | | | | | | Morning:  Evening: | | | | |
|  | What types of services does this facility provide? | | | | | | | | | | | 1. Family Planning 2. Antenatal Care 3. Postnatal Care 4. Post-abortion Care 5. Growth Monitoring 6. Child Immunization 7. Curative Services 8. Referral services | | | | |
|  | When was the last time a supervisor from outside this facility came here to visit? | | | | | | | | | | | 1. Never external supervision 2. Within the past 6 months 3. More than 6 months ago 4. Don’t know 5. No response | | | | |
|  | **INFORMATION REGARDING CATCHMENT AREA** | | | | | | | | | | | | | | | |
|  | Has the U-PHC identified the catchment population? | | | | | | | | | | | Yes/No  (IF NO THEN SKIP TO QUESTION No. 15) | | | | |
|  | Is the catchment population mapped and surveyed by the ANM in the last one year? | | | | | | | | | | | Yes/No | | | | |
|  | Do you have an estimate of the size of the catchment population that this facility serves that is, the target, or total population living in the area served by this facility? | | | | | | | | | | | Yes/No  (IF NO THEN SKIP TO QUESTION No. 15) | | | | |
|  | What is the size of the catchment population?  *Record the number of people living in the area served by this facility.* | | | | | | | | | | | Total Population:  Total No of Slums:  Slum Population: | | | | |
|  | Do you have an estimate of the no. of eligible couples in your catchment population? | | | | | | | | | | | Yes/No  (IF NO THEN SKIP TO QUESTION No. 15) | | | | |
|  | If yes, how many eligible couples are there? | | | | | | | | | | | Total:  Slum: | | | | |
|  | Are the secondary and tertiary centres identified for Family planning referral? | | | | | | | | | | | Yes/No | | | | |
|  | **INFRASTRUCTURE** *(to be observed)* | | | | | | | | | | | | | | | |
|  | Is the location of the U-PHC easily accessible? | | | | | | | | | | | Yes / No | | | | |
|  | What is the total space and work space allocation? | | | | | | | | | | | 1. Adequate 2. Inadequate | | | | |
|  | Are the building and compound in good condition? | | | | | | | | | | | Yes / No | | | | |
|  | Does it have a boundary wall and gate? | | | | | | | | | | | Yes / No | | | | |
|  | Does it have drinking water supply? | | | | | | | | | | | Yes / No | | | | |
|  | Does it have 24 hour electricity with backup facilities? | | | | | | | | | | | Yes / No | | | | |
|  | Does it have facilities for handicapped (e.g. ramp/wheelchairs)? | | | | | | | | | | | Yes / No | | | | |
|  | Does it have prominent display boards with signage:   1. Name of the U-PHC 2. Name of MO I/C 3. Family planning service availability in local language 4. Board with timings of U-PHC displayed 5. IEC material on Family planning | | | | | | | | | | | Yes / No  Yes / No  Yes / No  Yes / No  Yes / No (If yes, Specify) | | | | |
|  | Does it have a waiting area with:   1. Shelter 2. Adequate space 3. Benches for sitting 4. Walls with posters imparting Family planning education 5. Family planning Booklets / Leaflets provided 6. Separate toilet with adequate water supply | | | | | | | | | | | Yes / No  Yes / No  Yes / No  Yes / No  Yes / No  Yes / No | | | | |
|  | Does it have a separate room for OPD? | | | | | | | | | | | Yes / No  (IF NO SKIP TO QUESTION No. 27) | | | | |
|  | Does that OPD room have:   1. Light 2. Ventilation 3. Wash basin with water supply 4. Examination area curtained with adequate privacy | | | | | | | | | | | Adequate/Inadequate  Artificial/Sunlight  Adequate/Inadequate  Yes / No  Yes / No | | | | |
|  | Is there a separate room for providing Family planning counselling / services? | | | | | | | | | | | Yes / No  (IF NO SKIP TO QUESTION No. 29) | | | | |
|  | Does the room have:   1. Sufficient privacy 2. IEC material on family planning 3. Booklet/ leaflets on Family planning 4. Provisions of self service for condom distribution | | | | | | | | | | | Yes / No  Yes / No  Yes / No  Yes / No | | | | |
|  | Does it have a separate room for IUCD insertion? | | | | | | | | | | | Yes / No  (IF NO SKIP TO QUESTION No. 31) | | | | |
|  | Does that room have:   1. Space 2. Light 3. Ventilation 4. Has sufficient privacy/ curtains 5. Examination table 6. Wash basin with water supply | | | | | | | | | | | Adequate/Inadequate  Adequate/Inadequate  Artificial/Sunlight  Adequate/Inadequate  Yes / No  Yes / No  Yes / No | | | | |
|  | How many hand-washing facilities are available on site for staff to use? | | | | | | | | | | | Number of facilities:___________ | | | | |
|  | Ask to see the nearest hand washing facility. At the hand washing facility  OBSERVE:  *Select all that apply.*   1. Soap is present 2. Water source is present: stored water 3. Water source is present: running water 4. Hand washing area is near a sanitation facility | | | | | | | | | | | Yes / No  Yes / No  Yes / No  Yes / No | | | | |
|  | Observe the place where contraceptive supplies are stored and report on the following condition:   1. Is the Store space adequate? 2. Are all the methods off the floor? 3. Are all the methods protected from water? 4. Are all the methods protected from the sun? 5. Is the room clean of evidence of rodents (bats, rats) or pests (cockroaches…)? | | | | | | | | | | | Yes / No  Yes / No  Yes / No  Yes / No  Yes / No | | | | |
|  | **STAFFING PATTERN** | | | | | | | | | | | | | | | |
|  | Now I have some questions about staffing for this facility.  For the following questions, please tell me how many staff are currently assigned to this facility.  We want to know the age, sex, and duration of posting in the facility, technical qualification, training and refresher training of the staff members on family planning. | | | | | | | | | | | | | | | |
|  | **Staff** | **Age** | | | **Sex** | | | | **Duration of posting in the facility** | | | | **Qualification** | | | |
|  | Medical Officer 1 |  | | |  | | | |  | | | |  | | | |
|  | Medical Officer 2 |  | | |  | | | |  | | | |  | | | |
|  | PHN |  | | |  | | | |  | | | |  | | | |
|  | ANM 1 |  | | |  | | | |  | | | |  | | | |
|  | ANM 2 |  | | |  | | | |  | | | |  | | | |
|  | ANM 3 |  | | |  | | | |  | | | |  | | | |
|  | ANM 4 |  | | |  | | | |  | | | |  | | | |
|  | ANM 5 |  | | |  | | | |  | | | |  | | | |
|  | Pharmacist |  | | |  | | | |  | | | |  | | | |
|  | Store Keeper |  | | |  | | | |  | | | |  | | | |
|  | LT |  | | |  | | | |  | | | |  | | | |
|  | CDEO |  | | |  | | | |  | | | |  | | | |
|  | Dresser |  | | |  | | | |  | | | |  | | | |
|  | SMO |  | | |  | | | |  | | | |  | | | |
|  | NO |  | | |  | | | |  | | | |  | | | |
|  | SCC 1 |  | | |  | | | |  | | | |  | | | |
|  | SCC 2 |  | | |  | | | |  | | | |  | | | |
|  | SCC 3 |  | | |  | | | |  | | | |  | | | |
|  |  | | | | | | | | | | | | | | | |
|  | Staff | Training on OCP | | Training on Condoms | | | | Training on Cu-T | | Training on EC | | | Training on FP counselling | | Refresher Training in last 1 year (Specify) | |
|  | Medical Officer 1 |  | |  | | | |  | |  | | |  | |  | |
|  | Medical Officer 2 |  | |  | | | |  | |  | | |  | |  | |
|  | ANM 1 |  | |  | | | |  | |  | | |  | |  | |
|  | ANM 2 |  | |  | | | |  | |  | | |  | |  | |
|  | ANM 3 |  | |  | | | |  | |  | | |  | |  | |
|  | ANM 4 |  | |  | | | |  | |  | | |  | |  | |
|  | ANM 5 |  | |  | | | |  | |  | | |  | |  | |
|  | If Medical Officer and / or ANM is not trained for family planning services, ask for reason as to why he/she was not trained? | | | | | | | | | | |  | | | | |
|  | **FAMILY PLANNING SERVICES** | | | | | | | | | | | | | | | |
|  | How many days in a week are family planning services/products offered / sold here? | | | | | | | | | | | Number of days:________ | | | | |
|  | Which of the following methods of contraception are counselled, provided, referred and/or charged? | | | | | | | | | | | | | | | |
|  | **Methods of contraception** | | **Counselled**  **(Yes / No)** | | | **Provided (Number)** | | | | | **Referred**  **(Number)** | | | **Service Charged**  **(Yes / No)** | | **Incentive**  **(Yes / No)** |
|  | Female Sterilization | |  | | |  | | | | |  | | |  | |  |
|  | Male Sterilization | |  | | |  | | | | |  | | |  | |  |
|  | IUD | |  | | |  | | | | |  | | |  | |  |
|  | Injectables | |  | | |  | | | | |  | | |  | |  |
|  | Pill | |  | | |  | | | | |  | | |  | |  |
|  | Male Condom | |  | | |  | | | | |  | | |  | |  |
|  | Female Condom | |  | | |  | | | | |  | | |  | |  |
|  | Emergency Contraception | |  | | |  | | | | |  | | |  | |  |
|  | Diaphragm | |  | | |  | | | | |  | | |  | |  |
|  | Foam/Jelly | |  | | |  | | | | |  | | |  | |  |
|  | Std. Days/Cycle beads | |  | | |  | | | | |  | | |  | |  |
|  | LAM | |  | | |  | | | | |  | | |  | |  |
|  | Rhythm method | |  | | |  | | | | |  | | |  | |  |
|  | Withdrawal | |  | | |  | | | | |  | | |  | |  |
|  | MTP | |  | | |  | | | | |  | | |  | |  |
|  |  | | | | | | | | | | | | | | | |
|  | Do you have any information that out of the cases referred for sterilization how many were operated? | | | | | | | | | | | Yes / No  (IF NO SKIP TO QUESTION No.40) | | | | |
|  | If yes, specify? | | | | | | | | | | | Male:  Female: | | | | |
|  | Is there any special provision for providing family planning services to males? | | | | | | | | | | | Yes / No  (IF NO SKIP TO QUESTION No. 42) | | | | |
|  | If yes, then specify? | | | | | | | | | | |  | | | | |
|  | Do you conduct any family planning camps? | | | | | | | | | | | Yes / No  (IF NO SKIP TO QUESTION No.44) | | | | |
|  | If yes, frequency and timings? | | | | | | | | | | |  | | | | |
|  | Does ANM from the centre go for house to house visits? | | | | | | | | | | | Yes / No | | | | |
|  | Does she conduct RID / HND? | | | | | | | | | | | Yes / No | | | | |
|  | If Yes, How many times has she conducted HND in the last three months? | | | | | | | | | | |  | | | | |
|  | Was Family planning discussed in these HNDs? | | | | | | | | | | | Yes / No | | | | |
|  | Does she distribute contraceptives in these HNDs? | | | | | | | | | | | Yes / No | | | | |
|  | In the past 12 months, have there been any meetings where service statistics (or inventory) for family planning are discussed with staff? | | | | | | | | | | | Yes / No | | | | |
|  | **STOCK AND EQUIPMENTS** *(at the time of interview)*  *PF: Present and functional, PNF: Present BUT not functional, NP: Not Present* | | | | | | | | | | | | | | | |
|  | Does the U-PHC have the following stock?   1. ≥ 2 Cu T sets available 2. ≥ 10 Cu Ts are available 3. ≥ 10 pairs of sterile gloves are available 4. 30 cycles of OCs are available 5. 10 E-pills packs are available 6. 200 pieces of condoms available | | | | | | | | | | | PF / PNF / NP  PF / PNF / NP  PF / PNF / NP  PF / PNF / NP  PF / PNF / NP  PF / PNF / NP | | | | |
|  | Does this facility have the following supplies needed to insert and/or remove IUDs?   1. Sponge holding forceps 2. Speculums (Large and medium) 3. Scissors 4. Anterior vaginal wall retractor 5. Valselum 6. Uterine sound 7. Sealed IUD pack 8. Clean gloves 9. Cotton swabs 10. Antiseptic 11. Antibiotic | | | | | | | | | | | PF / PNF / NP  PF / PNF / NP  PF / PNF / NP  PF / PNF / NP  PF / PNF / NP  PF / PNF / NP  PF / PNF / NP  PF / PNF / NP  PF / PNF / NP  PF / PNF / NP  PF / PNF / NP | | | | |
|  | Are the equipment’s available as per standard? | | | | | | | | | | | Yes / No | | | | |
|  | Are the services guidelines/ norms for procedures available? | | | | | | | | | | | Yes / No | | | | |
|  | Is there a regular indent/ supply system? | | | | | | | | | | | Yes / No | | | | |
|  | When did you last replenish your stock of contraceptives? | | | | | | | | | | |  | | | | |
|  | How often are your stocks replenished? | | | | | | | | | | | 1. In Days 2. In Weeks 3. In Months | | | | |
|  | You mentioned that you typically provide the [METHOD] at this facility; can you show it to me?  If no, probe: Is the [METHOD] out of stock today? | | | | | | | | | | | 1. In-stock 2. Out of stock   (IF IN STOCK THEN SKIP TO QUESTION No. 60) | | | | |
|  | How many days has the [METHOD] been out of stock? | | | | | | | | | | |  | | | | |
|  | Has the [METHOD] been out of stock at any time in the last 3 months? | | | | | | | | | | | Yes / No | | | | |
|  | **RECORDS AND REGISTERS** | | | | | | | | | | | | | | | |
|  | Are the following records available at the U-PHC?   1. Household survey register available 2. Eligible couple register available 3. Family planning register available 4. Referral register | | | | | | | | | | | Yes / No  Yes / No  Yes / No  Yes / No  (IF NO END INTERVIEW) | | | | |
|  | From the registers, record: *(in the last three months)* | | | | | | | | | | | | | | | |
|  | **Method** | | | | | | **Distributed at centre** | | | | | | | | **Distributed in the field** | |
|  | Male condom | | | | | |  | | | | | | | |  | |
|  | OCP | | | | | |  | | | | | | | |  | |
|  | IUD | | | | | |  | | | | | | | |  | |
|  | Injectable | | | | | |  | | | | | | | |  | |
|  | Emergency contraceptive | | | | | |  | | | | | | | |  | |
|  |  | | | | | | | | | | | | | | | |
